# Comparison of enteral prucalopride *versus* intravenous metoclopramide for feeding intolerance in patients with critical illness: a randomized double-blinded study

**DOI:** 10.3389/fphar.2024.1413246

**Published:** 2024-11-08

**Authors:** Eman Mohamed Elmokadem, Dina Khaled Abou El Fadl, Nermin Eissa, Najla Abdulaziz Alnassar, Ahmed M. Bassiouny, Amir Eskander Hanna Samy, Nouran Omar El Said

**Affiliations:** ^1^ Department of Pharmacy Practice and Clinical Pharmacy, Faculty of Pharmacy, Future University in Egypt, Cairo, Egypt; ^2^ Department of Biomedical Sciences, College of Health Sciences, Abu Dhabi University, Abu Dhabi, United Arab Emirates; ^3^ Human Nutrition and Dietetics, College of Health Sciences, Abu Dhabi University, Abu Dhabi, United Arab Emirates; ^4^ Faculty of Medicine, Ain Shams University, Cairo, Egypt; ^5^ Critical Care Department, El Matarya Teaching Hospital, Cairo, Egypt

**Keywords:** prokinetic, feeding intolerance, gastric residual volume, prucalopride, critical illness

## Abstract

**Background:**

Feeding intolerance is commonly experienced during enteral feeding, necessitating cessation. Metoclopramide may be given to assist gastric emptying, but patients experience adverse effects and gradual loss of efficacy. Prucalopride, a safer prokinetic, may play a role in gastric emptying. Therefore, the current study aimed to assess its effectiveness and safety in feeding intolerance developed in critically ill patients.

**Materials and Methods:**

In this prospective randomized double-blinded study, patients with feeding intolerance were randomized to receive 2 mg prucalopride enterally once daily or 10 mg metoclopramide intravenously every 6–8 h for 7 days. Patients were monitored for treatment failure, successful feeding, gastric residual volume (GRV), and the development of medication-related adverse effects.

**Results:**

A total of 70 patients (35 in the metoclopramide group and 35 in the prucalopride group) completed the study. The average daily GRV in the prucalopride group was significantly lower compared to the metoclopramide group (p=<0.001) on day 7. Additionally, the percentage change in GRV from day 1 to day 7 showed a greater significant change in the prucalopride arm *versus* the metoclopramide arm (p=<0.001). The treatment groups were comparable in terms of ICU length of stay (*p* = 0.094). Moreover, there was a significantly higher successful caloric intake in the prucalopride group compared to the metoclopramide group on day 7 (*p* = 0.039).

**Conclusion:**

Prucalopride administration in enterally fed patients with feeding intolerance may reduce GRV and improve feeding success rates compared to metoclopramide treatment. The use of prucalopride was found to be tolerable and safe in critically ill patients.

**Clinical Trial Registration:**

clinicaltrials.gov, identifier NCT05496179

## 1 Introduction

Prolonged hospitalization of critically ill patients in the Intensive Care Unit (ICU) has validated the significance of receiving nutritional support. This is due to severe catabolism experienced and their need for continuous life-maintaining treatment (Hoffer and Bistrian, 2016). Enteral nutrition (EN) is a proactive therapeutic approach to avoiding malnutrition complications and improving critically ill patient outcomes (McClave et al., 2016). However, feeding intolerance frequently develops during EN, which might result in adjustments or the discontinuation of EN (McClave et al., 2020).

The definition of feeding intolerance is not fully understood; however, clinical symptoms include vomiting, a significant gastric residual volume (GRV), diarrhea, and abdominal distension (Blaser et al., 2014; Reintam Blaser et al., 2015). Gastric residual volume is frequently used as a surrogate measure for feeding intolerance in clinical practice (Metheny et al., 2012; Elke et al., 2015). Research has linked feeding intolerance with lengthy ICU stays and mortality risks therefore, it should be aggressively treated (Blaser et al., 2014; Gungabissoon et al., 2015; Hu et al., 2020).

One of the major contributing factors to feeding intolerance is delayed gastric emptying. Drugs can therefore be used to help critically ill patients’ tolerance and promote gastric emptying (Deane et al., 2007) Treatment with prokinetics, such as metoclopramide, is a frequently utilized approach in the management of feeding intolerance. However, it is associated with adverse drug effects (ADEs) on the central nervous system (CNS), including motor disorders and cardiovascular side effects such as QT prolongation (Lewis et al., 2016; Peng, 2020). Moreover, after a few days of therapy, the drug’s effects start to diminish and fade (Jolliet et al., 2007; van der Meer et al., 2014).

Additionally, prokinetics such as cisapride and erythromycin are correlated with QT prolongation and cardiac arrhythmias (MacLaren et al., 2001; Nguyen et al., 2011). Due to these safety concerns with the currently available prokinetics, researchers are looking for prokinetic alternatives with comparable or higher effectiveness and a better tolerance profile.

Prucalopride is a third-generation, selective 5-hydroxytryptamine 4 (5-HT4) receptor agonist that increases the rate of gastric emptying and is used in constipation (Carbone et al., 2019). According to recent research, prucalopride may be valuable in treating several motility problems, including gastroparesis, by increasing gastric emptying and promoting gastric motility (Hong, 2021). Prucalopride was found to enhance gastrointestinal transit in healthy volunteers, canine models, and individuals with persistent constipation (Prins et al., 2001; Camilleri and Atieh, 2021). Moreover, its high selectivity and reduced potential for developing cardiac adverse effects make it an appropriate and safer candidate (Mendzelevski et al., 2012). Therefore, clinical trials are required to study its effect on gastroparesis treatment and feeding intolerance. The current study aims to examine the efficacy and safety of the use of prucalopride in comparison to metoclopramide on the gastric residual volume in enterally fed critically ill patients with delayed gastric emptying.

## 2 Materials and methods

### 2.1 Study design and setting

This study was a prospective randomized double-blinded trial conducted in accordance with the Declaration of Helsinki from August 2022 to June 2023 at the ICU of El-Mataria Teaching Hospital, Cairo, Egypt.

### 2.2 Ethical considerations

Ethical approval was granted from the Research Ethics Committee for Experimental and Clinical Studies, Faculty of Pharmacy, Future University in Egypt (REC-FOPFUE-16/126) and was registered in clinicaltrials.gov (NCT05496179). Informed consent was obtained from all participants or caregivers before joining the study.

### 2.3 Methodology

#### 2.3.1 Study population

Patients (males or females) presenting to the ICU having delayed gastric emptying with an expected minimum length of ICU stay of a week were screened for eligibility.

Patients aged 18–60 years, receiving enteral tube feeding, diagnosed with enteral feeding intolerance (EFI), and presenting with a modified nutritional risk in the critically ill (mNUTRIC) score of ≥5 were enrolled in the study. EFI was defined as either at least a single measurement of GRV of ≥250 mL as assessed by ultrasonography or gastrointestinal symptoms (nausea, vomiting, diarrhea, and abdominal distension) developed during enteral feeding. Despite the variation in GRV definitions in diagnosing EFI, this particular GRV threshold was chosen based on previous reports (Blaser et al., 2014; Charoensareerat et al., 2021).

Patients were excluded if they had a known hypersensitivity to prucalopride or metoclopramide, prior prokinetic use within 48 h or recent GI surgery, GI obstruction, gastroparesis (clinically diagnosed), bleeding or perforation, history of gastrectomy or esophagectomy, acute CNS infection or injury.

Exclusion criteria also included obesity, pregnancy, hemodynamic instability, presence of cardiac arrhythmia, prolonged QT interval, and diabetes. Patients with clinically significant renal or hepatic impairment or with an estimated short extubation time of less than 48 h were also excluded.

#### 2.3.2 Study intervention

Eligible patients were randomly assigned to either of the two study groups at a 1:1 ratio using a software-generated list of random numbers. Participant randomization assignment was then kept in sealed, signed envelopes. A designated team member not involved in patient care or data collection used this sequence to allocate treatments. The randomization key was securely stored and only accessible to this team member. Patients, clinicians, radiologists, and unit staff responsible for assessments were blinded from group allocation. Additionally, all other operating personnel staff undergoing lab analysis and plasma collection were blinded to group assignment. This blinding was maintained throughout the study period and during initial data analysis.

The Prucalopride group received 2 mg prucalopride enterally once daily, and the metoclopramide group received 10 mg metoclopramide intravenously every 6–8 h. Participants were treated and followed up for 7 days.

##### 2.3.2.1 Enteral feeding protocol

All the participants received standard feeding formula as Fresubin^®^ (Fresenius Kabi, Egypt) with 1.5 kcal/mL caloric density, administered continuously through a nasogastric tube. The feeding was started at a rate of 20 mL/h and steadily increased until the patient received their daily caloric target requirement. The daily caloric target needs were calculated according to guidelines as 25 kcal/kg/d, and the protein requirement was 1.4 g/kg/day (McClave et al., 2016; Singer et al., 2019).

Treatment failure necessitating feeding discontinuation was defined as patients who developed GI symptoms of feeding intolerance, such as nausea, vomiting, diarrhea, and abdominal distension, for two consecutive incidents despite low feeding rates. Experiencing a study medication-related intolerable or severe adverse effects, as well as the need to terminate tube feeding or discharge from ICU, warranted a patient’s departure from the study.

#### 2.3.3 Study procedure

Patient Data Collection: At baseline, data collected included demographic data, weight, height, body mass index (BMI), ICU admission cause and date, disease severity as assessed by the Acute Physiology and Chronic Health Evaluation II (APACHE II) (Knaus et al., 1985) and organ function as evaluated by the Sequential Organ Failure Assessment (SOFA) (Vincent et al., 1998). Medical history, complete medication history, and nutrition data (daily caloric target and daily received calories) for each patient were also recorded.

Clinical Assessment: Complete physical cardiovascular, respiratory, and neurological examinations were performed. An abdominal examination was also conducted to detect signs of feeding intolerance, and the mNUTRIC score was calculated (Brascher et al., 2020).

Assessment of GRV: Radiological assessment of GRV was measured using a “GE LOGIQ E9″ 2-dimensional ultrasound device at 4 hrs. Intervals, performed by a single-blinded assessor as previously reported (Elmokadem et al., 2021). Participants were asked to lie on their right side half an hour after feeding. Following this, an ultrasound examination was conducted to measure the cross-sectional area (CSA) of the stomach’s antrum region. To determine the GRV, researchers applied a pre-validated mathematical equation: GRV (mL) = 27 + 14.6 × right-lat CSA - 1.28 × age (Perlas et al., 2013).

Laboratory investigations: Lab investigations included complete blood count, liver enzymes, serum creatinine, lipid profile as well as fasting blood glucose.

Monitoring and follow-up: Patients were also monitored for the occurrence of vomiting and/or feeding intolerance and the development of adverse drug events such as abdominal pain and QT prolongation.

#### 2.3.4 Study outcomes

The primary outcome of the present study was average gastric residual volume measured by ultrasonography and recorded at three-time points: baseline, midpoint, and at the end of the study.

The secondary outcomes included length of ICU stay as well as achieved percentage of caloric intake from target caloric needs and incidence of successful caloric intake at the midpoint and the end of the study. Successful caloric intake was defined as achieving a minimum of 80% of the target caloric intake.

### 2.4 Sample size measurement, data management, and analysis

The sample size was estimated using the NQuery statistical package, version 7.0, Los Angeles, CA. In the study by Elmokadem et al., the difference between prokinetics itopride and metoclopramide in the percent change in GRV was 18.4% with a pooled standard deviation of 18.9% (Elmokadem et al., 2021). Based on these findings, a minimal sample size of 29 subjects in each group is required at an alpha level of 0.05 and power of 90%. To overcome potential loss due to dropouts, the sample size was further increased to 37 subjects per group and a total sample size of 74 subjects.

Data management and statistical analysis were performed using the IBM SPSS Statistics for Windows, Version 22.0. IBM Corp., Armonk, NY. The normality of data distribution was assessed using the Kolmogorov-Smirnov test and Shapiro-Wilk test.

For continuous data, parametric data were summarized as means and standards of deviation, while non-parametric data were summarized as medians and interquartile ranges. Categorical data were summarized as percentages (%) and counts (n). Comparison between 2 groups for normally distributed numerical values was done using the unpaired Student’s t-test, while for non-normally distributed numerical values was done using the Mann-U Whitney test. For categorical data, Chi-squared and Fisher’s exact test were used to compare between groups. *p*-values <0.05 were considered significant.

## 3 Results

**FIGURE 1:**
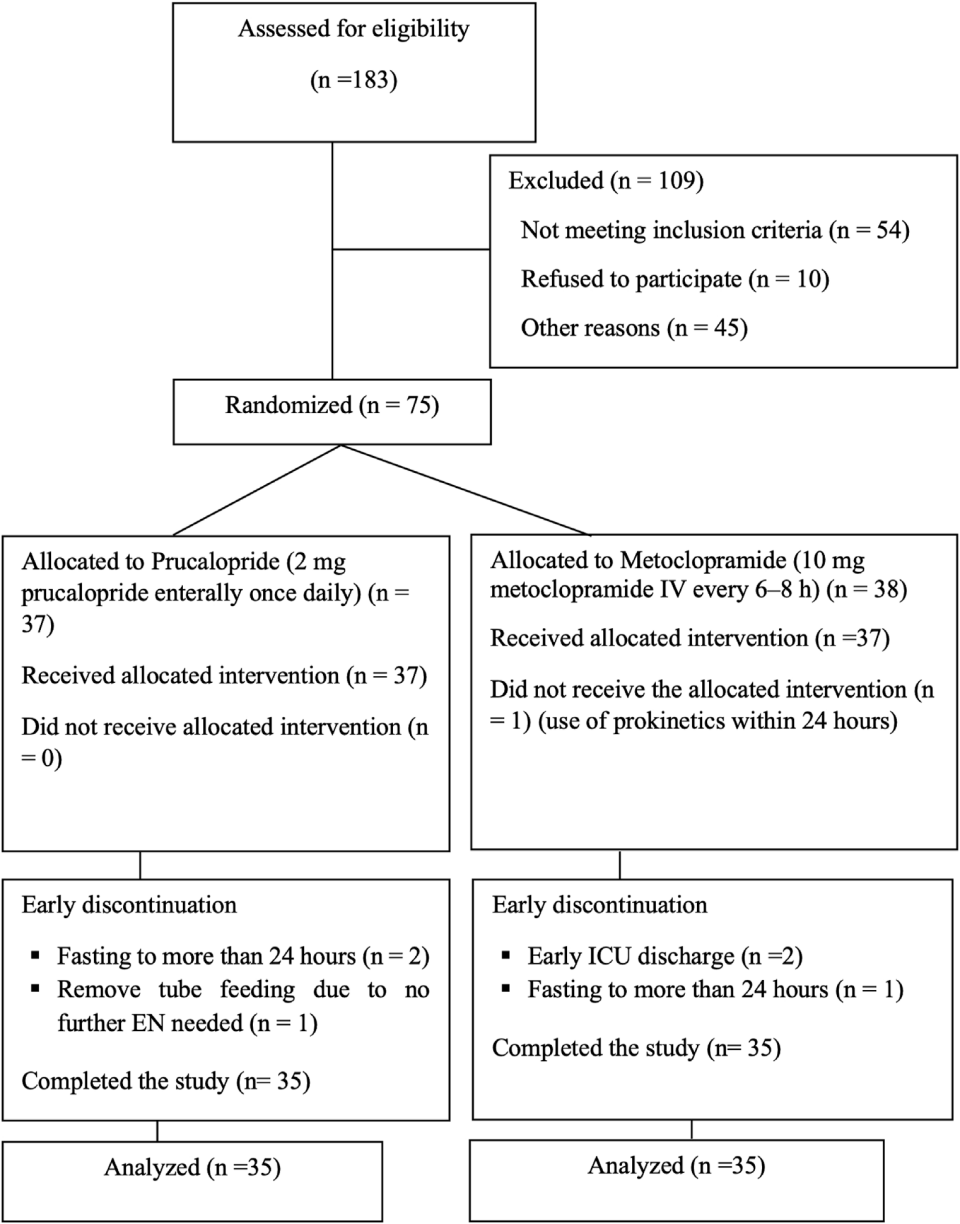
Study flow diagram.

A total of 183 patients presenting to the ICU from August 2022 to June 2023 were screened for inclusion; 75 were randomized, but only 70 patients remained after early withdrawal from study the study, as shown in Figure 1. The remaining analyzed subjects completed the 7 days of enteral feeding: 35 patients in the prucalopride and metoclopramide arm.

### 3.1 Baseline results

At baseline, the patients’ demographic, biochemical, and clinical parameters were comparable across both groups except for the hemoglobin levels, where the metoclopramide group showed significantly higher concentration compared to the prucalopride-treated group (9.86 ± 1.86 mg/dL and 8.51 ± 1.48 mg/dL, respectively, *p* = 0.01). Both study groups showed non-significant differences in the medication used, which included mainly opiates, sedatives, and vasopressors. Furthermore, the reasons for admission to the ICU were comparable in both groups (Table 1).

**TABLE 1:** Baseline demographic and clinical parameters.

Parameter	Metoclopramide (n = 35)	Prucalopride (n = 35)	*p*-value
Age (years); mean ± S.D	46.86 ± 11.67	45 ± 11.41	0.55^a^
Gender; n (%)			
Male	19 (54.3)	14 (40)	0.231^c^
Female	16 (45.7)	21 (60)	
BMI(kg/m2); mean ± S.D	23.38 ± 4.3	25.35 ± 5.9	0.115^a^
APACHE II; mean ± S.D	19.74 ± 3.56	20.01 ± 3.424	0.754^a^
SOFA; median [IQR]	9 [8–10]	8 [7–9]	0.093^b^
Modified NUTRIC score; median [IQR]	6 [5–7]	6 [5–6]	0.502^b^
Blood Chemistry			
Hb (gm/dL); mean ± S.D	9.86 ± 1.86	8.51 ± 1.478	0.01^a^ ^,^*
WBC (x10^9^/L); mean ± S.D	7.04 ± 1.23	6.4425.± 1.68	0.095^a^
BUN (mg/dL) mean ± S.D	23.89 ± 5.42	25.87 ± 4.43	0.098^a^
Serum Creatinine (mg/dL); median [IQR]	0.89 [0.8–0.93]	0.91 [0.77–1.11]	0.521^b^
AST (IU/L); mean ± S.D	29 ± 4.87	26.8 ± 6.22	0.104^a^
ALT (IU/L); mean ± S.D	28.3 ± 5.17	27.2 ± 4.75	0.403^a^
Albumin (g/dL); mean ± S.D	4.12 ± 0.21	4.32 ± 0.36	0.104^a^
Bilirubin (mg/dL); mean ± S.D	0.79 ± 0.087	0.78 ± 0.06	0.547^a^
FBG (mg/dL); mean ± S.D	84.85 ± 15.83	87.57 ± 15.33	0.528^a^
TC (mg/dL); mean ± S.D	144.7 ± 23.37	165.52 ± 29.35	0.127^a^
Medication; n (%)			
Opiates	8 (22.9)	10 (28.6)	0.584^c^
Benzodiazepine	12 (34.3)	9 (25.7)	0.434^c^
Inotropes/vasopressors	6 (17.1)	8 (22.9)	0.55^c^
Sedatives	6 (17.1)	5 (14.2)	0.743^c^
Paralytic agents	3 (8.6)	3 (8.6)	n.s.^d^
Reason for ICU admission; n (%)			
Medical	19 (54.29)	17 (48.71)	0.564^c^
Respiratory	8 (22.86)	7 (20)	0.771^c^
Neurologic	2 (5.71)	1 (2.86)	n.s.^d^
Burns	8 (22.86)	7 (20)	0.771^c^
Other	1 (2.86)	2 (5.71)	n.s.^d^
Surgical	16 (45.71)	18 (51.43)	0.632
Trauma	9 (25.71)	11 (31.43)	0.597^c^
Neurologic	3 (8.57)	2 (5.71)	n.s.^d^
Vascular	2 (5.71)	3 (8.57)	n.s.^d^
Other	2 (5.71)	2 (5.71)	n.s.^d^

BMI, body mass index; SOFA, sequential organ failure assessment; APACHE II, Acute Physiology and Chronic Health Evaluation II; NUTRIC, nutrition risk in critically ill; Hb, hemoglobin; WBC, white blood cells; BUN, blood urea nitrogen; AST, aspartate transaminase; ALT, alanine transaminase; FBG, fasting blood glucose; TC, total cholesterol.

Data are presented as mean ± SD:standard deviation or median [IQR: interquartile range].

*p-value ≤0.05 is considered significant.

Statistical Tests.

^a^ unpaired Student’s t-test.

^b^ Mann-Whitney test.

^c^ χ2test.

^d^ Fisher Exact.

### 3.2 Primary outcomes

The primary outcome (shown in Table 2), average daily GRV was non-significantly different between both the metoclopramide and prucalopride arms on days 1 and 4 (*p* = 0.426 and 0.253, respectively). Additionally, the percentage change between days 1 and 4 in gastric residual volume was comparable between both groups (*p* = 0.052). On day 7, the average daily GRV in the prucalopride group was significantly lower compared to the metoclopramide group (p=<0.001). Moreover, the percentage change in GRV between days 1 and 7 showed a greater significant change in the prucalopride arm *versus* the metoclopramide arm (*p* = <0.001).

**TABLE 2:** Change in average daily gastric residual volume.

	Metocolopramide (n = 35)	Prucalopride (n = 35)	*p*-value^a^	Mean difference (95% CI)
Average GRV, day1 (mL); mean ± S.D	348.56 ± 67.5	337.16 ± 50.33	0.426	−11.4 (-39.8,16.99)
Average GRV, day 4 (mL); mean ± S.D	260.14 ± 39.45	245.6 ± 63.24	0.253	−14.54 (-10.6,39.69)
Average GRV, day 7 (mL); mean ± S.D	207.70 ± 32.9	134.58 ± 72.7	<0.001*	−73.12 (-100, −46.2)
Percentage change in GRV (day 4,1); mean ± S.D	−22.65 ± 6.93	−28.68 ± 16.49	0.052	6.029 (-0.052, 12.064)
Percentage change in GRV (day 7,1); mean ± S.D	−38.15 ± 16.43	−59.12 ± 22.87	<0.001*	−20.97 (−30.49,-11.47)

GRV: gastric residual volume.

Data are presented as mean ± SD:standard deviation.

95% confidence interval is reported as (lower limit, upper limit).

*p-value ≤0.05 is considered significant.

Statistical Tests.

^a^ unpaired Student’s t-test.

### 3.3 Secondary outcomes

Secondary outcomes are demonstrated in Table 3. There was no significant difference between the treatment groups in terms of ICU length of stay (*p* = 0.094). When comparing the median daily percentage caloric intake, there was no significant difference between both groups on day 4 (*p* = 0.053). Percentage caloric intake increased across both groups, and a significant difference was recorded on day 7, with higher intake in the prucalopride-treated patients compared to patients receiving metoclopramide (84% and 80%, respectively, *p* = 0.01). The incidence of successful caloric intake was comparable between both groups on day 4 (*p* = 0.056) but with significantly higher successful caloric intake in the prucalopride group, compared to the metoclopramide group on day 7 (80% and 57.1%, respectively, *p* = 0.039).

**TABLE 3:** Secondary outcomes.

	Metocolopramide (n = 35)	Prucalopride (n = 35)	*p*-value
ICU length of stay	15 [13–16]	13 [12–15]	0.094^a^
Percentage of enteral caloric intake (day 4); median [IQR]	69 [63.2–72.6]	80 [71–78.3]	0.053^a^
Successful caloric intake (day 4); n (%)	14 (40)	22 (62.9)	0.056^b^
Percentage of enteral caloric intake (day 7); median [IQR]	80 [72–79.1]	84 [80–85]	0.01^a^*
Successful caloric intake (day 7); n (%)	20 (57.1)	28 (80)	0.039^b^*

ICU: intensive care unit.

Data are presented as median [interquartile range].

*p-value ≤0.05 is considered significant.

Statistical Tests.

^a^ Mann-Whitney test.

^b^ χ2test.

### 3.4 Safety

**FIGURE 2:**
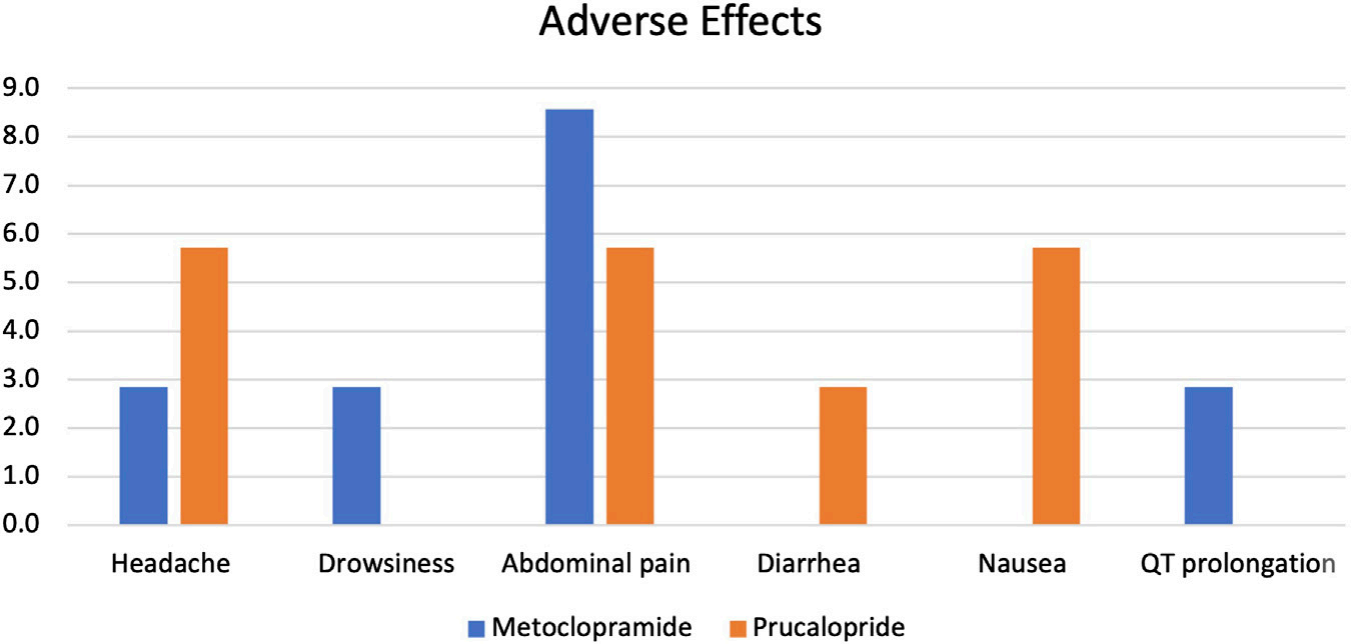
Adverse drug effects (%) reported in metoclopramide and prucalopride patients.

The proportion of adverse effects reported in both patient groups was non-significantly different, as shown in Figure 2. Minor adverse effects were reported in both groups, including headache, drowsiness, and abdominal pain. Only one case of QT prolongation was reported in the metoclopramide group, while in the prucalopride arm, one patient experienced diarrhea, and two patients experienced nausea. All reported adverse effects were mild and temporary and did not demand treatment discontinuation. Specifically, headaches typically lasted 2–4 h and were described as mild to moderate in intensity. Drowsiness was generally reported within the first 24–48 h of treatment initiation and subsided thereafter. Abdominal pain, when present, was described as mild discomfort that resolved within 1–2 days without intervention. The average duration of these side effects was 2.5 days, with no single effect lasting longer than 5 days in any patient. No severe or persistent adverse effects were observed throughout the study period.

## 4 Discussion

Almost half of critically ill patients suffer subsequent undernutrition, primarily caused by enteral feeding intolerance (Martindale et al., 2009; Araújo-Junqueira and De-Souza, 2012). Most of them suffer from reduced gastric emptying, and one-third of enteral interruption time is caused by enteral intolerance (Martindale et al., 2009; Btaiche et al., 2010; Ukleja, 2010; Araújo-Junqueira and De-Souza, 2012).

Prokinetics may therapeutically enhance EN tolerability, which suggests their promising off-label use in enhancing gastric emptying in critically ill patients (Pittayanon et al., 2018).

Metoclopramide is a centrally-acting antiemetic agent that increases gastric motility through muscarinic receptors (van Zanten, 2016). Intravenous administration of metoclopramide is frequently used to manage delayed gastric emptying and facilitate early enteral feeding (Baradari et al., 2017).

Metoclopramide and erythromycin have become the standards for treating EFI patients in most ICUs; however, their side effects restrict their clinical usefulness. As a result, patients with high risks of aspiration and gastric motility dysfunction still require new agents to improve feeding performance (van Zanten, 2016).

Prucalopride is approved by the European Medicines Agency (EMA) and the FDA for the treatment of chronic constipation (FDA, 2018; Mahajan, 2019). Recent evidence indicates that prucalopride may enhance gastric motility and improve gastric emptying and thus can be used in several motility problems, including the management of gastroparesis (Hong, 2021). However, this is the first study to investigate the impact of prucalopride on enteral feeding intolerance.

### 4.1 Primary outcome

In the current study, a significant difference was observed between prucalopride and metoclopramide regarding the change in GRV on day seven compared to baseline, however, on day four, the GRV change was comparable between both groups. This suggests that prucalopride may be superior to metoclopramide in reducing GRV and improving gastric emptying.

The significant difference in GRV change detected on day seven can be explained by the development of tachyphylaxis in patients receiving metoclopramide. A previous randomized trial has shown that the effect of metoclopramide on ameliorating feed intolerance in critical illness dropped 56% by day 3% and 74% by the seventh day. Several mechanisms were proposed to explain the diminished responses over time. These include a reduced sensitivity of receptor cells, a decrease in the number of active receptors, and the endocytosis of receptors. (Nguyen et al., 2007).

Unlike with metoclopramide use, tachyphylaxis was not reported with prucalopride in the current study since the study period was only 7 days. The short period failed to demonstrate the gradual loss of beneficial effects of prucalopride previously recorded only after the first few weeks of treatment for constipation, indicating the need for longer treatment time to develop tolerance (Hong, 2021).

Our findings agree with those of a randomized cross-over study, where prucalopride significantly improved gastric emptying time compared to placebo in patients with gastroparesis (Carbone et al., 2019). Moreover, patients experienced a notable improvement in symptoms affecting the upper gastrointestinal tract (nausea, vomiting, and reflux) when treated with prucalopride, as opposed to when they received a placebo. Symptoms associated with eating, particularly feelings of fullness after meals and bloating, were significantly reduced when patients were treated with prucalopride compared to when they received a placebo.

Another study by Andrews CN et al. exhibited a more rapid gastric emptying in diabetic and connective tissue-related gastroparesis patients receiving prucalopride compared to placebo (Andrews et al., 2021). This preliminary study did not demonstrate an improvement in symptoms when using 4 mg of prucalopride in patients primarily suffering from diabetic gastroparesis. However, the drug did significantly accelerate gastric emptying in these patients.

A single-centered phase 2a study evaluated the effectiveness of a selective agonist of 5-HT4 receptors, TAK-954, IV infusion against IV metoclopramide (10 mg given four times daily) in 13 critically ill patients experiencing EFI, as evidenced by high GRV. The results showed that a higher percentage of patients treated with TAK-954 achieved normal gastric retention and improved gastric emptying compared to those given metoclopramide. The reported findings support our inferences on the effectiveness of 5-HT4 receptor agonists in EFI(Chapman et al., 2021).

A previous clinical study compared the effects of a 5-HT4 receptor agonist (cisapride) with metoclopramide in critically ill patients. A total of 14 patients with high gastric residual volumes received either 10 mg of enteral cisapride or 10 mg of metoclopramide for up to a week (15). While both medications improved gastric motility, metoclopramide was more effective in reducing gastric residual volumes. However, this difference did not translate to clinical significance, as both drugs allowed patients to reach similar maximum feeding rates. In contrast, the current study suggests that prucalopride, a selective 5-HT4 receptor agonist, may be more effective than metoclopramide as a prokinetic agent. This differs from the earlier findings with cisapride, which is a non-selective 5-HT4 receptor agonist.

Other prokinetic agents also appear to have positive effects on gastrointestinal function and feeding tolerance (Peng et al., 2021). A previous study reported a significant improvement in GRV when using a combination of IV metoclopramide and IV neostigmine compared to patients using a monotherapy of any of them (Baradari et al., 2017).

Similarly, the findings of another study revealed that neostigmine resulted in GRV improvement in more patients compared to metoclopramide (Rahat-Dahmardeh et al., 2021). Furthermore, a combination of IV metoclopramide and enteral erythromycin showed significantly lower daily accumulative GRV compared to placebo (Charoensareerat et al., 2021).

Additionally, when comparing the effect of itopride to metoclopramide, itopride similarly demonstrated a significant decrease in GRV at day 7, with a higher percentage of GRV change compared to baseline (Elmokadem et al., 2021). This may further confirm the temporal decline in the effect of metoclopramide compared to other prokinetic drugs.

Despite the demonstrated effect of prokinetics, some earlier studies failed to show a positive effect of metoclopramide in improving clinical outcomes in patients with feeding intolerance (Marino et al., 2003; Nursal et al., 2007; Dickerson et al., 2009; Acosta-Escribano et al., 2014).

### 4.2 Secondary outcomes

Increased successful feeding (>80% of feeding goal) correlated with reduced hospital stay and mortality rates (Martin et al., 2004).

The findings of the current study recorded a significant difference in the daily percentage of caloric intake in favor of the prucalopride-treated patients. The incidence of successful caloric intake was non-significantly different between both groups on day four but significantly higher in the prucalopride group compared to the metoclopramide group on day 7.

Additionally, a significant difference in the total daily energy intakes was only reported on day six among patients receiving a combination of metoclopramide and erythromycin compared to patients using a monotherapy of metoclopramide (Charoensareerat et al., 2021).

Conversely, Nguyen et al. reported that successful feeding incidence among patients receiving metoclopramide progressively declined throughout days 1–7 (Nguyen et al., 2007).

Moreover, a previous study reported that nasointestinal (NI) feeding increased the successful feeding goal percentage when compared to nasogastric (NG) feeding in addition to a prokinetic combination (metoclopramide and erythromycin) treatment on most of the days, particularly in days 4 and 5 (Taylor et al., 2016). These findings further emphasize the tachyphylaxis phenomenon associated with the administration of metoclopramide.

Furthermore, Heyland et al. reported that the use of prokinetic ulimorelin was comparable to metoclopramide use, increasing feeding success and the percentage of daily protein prescribed across the 5 days of treatment (Heyland et al., 2019). This aligns with our findings regarding the observation of a significant difference in successful caloric intake on day seven but not before.

The current study showed comparable length of stay in ICU across the treatment groups. Similarly, earlier studies also failed to demonstrate a subsequent reduction in the length of stay in the ICU with the use of prokinetic drugs (Heyland et al., 2019; Charoensareerat et al., 2021; Cheng et al., 2021). This indicates the lack of conclusive evidence that prokinetics may improve clinical outcomes such as length of stay in critically ill patients due to the limited sample size.

### 4.3 Safety

The findings of the current study reported minor adverse effects in both groups, including headache, drowsiness, and gastrointestinal symptoms that did not require treatment discontinuation. However, QT prolongation was reported only in the metoclopramide group. This is in accordance with data from a systematic review, which revealed that prucalopride was generally well-tolerated, with temporary nausea, headache, and diarrhea being the initial adverse events most commonly reported (Ali et al., 2021). Comparable results were also reported in previous studies (Ke et al., 2012; Yiannakou et al., 2015; Camilleri et al., 2016).

Similar to our results, cardiovascular side effects were reported with metoclopramide treatment in several previous studies (Baradari et al., 2017; Heyland et al., 2019; Charoensareerat et al., 2021; Rahat-Dahmardeh et al., 2021).

The effects of prucalopride in individuals with mental health conditions who are taking multiple antidepressants or antipsychotic medications require additional study. This is particularly important due to prucalopride’s ability to stimulate 5-HT4 receptors. Interestingly, animal study results indicate that combining a selective serotonin reuptake inhibitor (SSRI), citalopram, with prucalopride could potentially enhance the rapid antidepressant effects of the 5-HT4 agonist (Lucas et al., 2010). However, in the critical care setting, co-administration of two serotonergic medications may increase the risk of developing the life-threatening but under-reported condition known as serotonin syndrome (Prakash et al., 2021; Miller et al., 2023).

### 4.4 Study limitations

The strengths of this study were that treatments were blinded and randomized, and all participants received a standard feeding formula.

The study’s limitations were that it was not placebo-controlled and included a small sample size, which failed to demonstrate the effect of prucalopride in secondary clinical outcomes. Larger cohorts are recommended to further investigate the efficacy of prucalopride for these outcomes.

## 5 Conclusion

Results of the current study suggest that prucalopride use in enterally fed patients with feeding intolerance may significantly reduce GRV and improve feeding success rates and caloric intake compared to metoclopramide treatment but with no effect on ICU length of stay. The use of prucalopride was found to be safe and tolerable in critically ill patients. These findings require further investigation with a larger sample to adequately evaluate the impact of prucalopride treatment on secondary clinical outcomes and to verify the reported conclusions.

## Data Availability

The raw data supporting the conclusions of this article will be made available by the authors, without undue reservation.
